# Supplementary material for: De Novo Design of Miniprotein Inhibitors of Bacterial Adhesins
Source: bioRxiv. 2025 Aug 18:2025.08.18.670751. Preprint. [Version 1] doi: 10.1101/2025.08.18.670751 (PMC12393372; doi:10.1101/2025.08.18.670751)
Supplement: Supplement 1 [file NIHPP2025.08.18.670751v1-supplement-1.pdf]

bioRxiv preprint doi: https://doi.org/10.1101/2025.08.18.670751; this version posted August 18, 2025. The copyright holder for this preprint (which was not certified by peer review) is the author/funder, who has granted bioRxiv a license to display the preprint in perpetuity. It is made available under aCC-BY 4.0 International license.

Supplemental Figures

Supplementary Table 1. Data collection and refinement statistics for crystal structures.

	F7-FimH (PDB Code: 9Q1V)	A7-Abp2D (PDB Code: 9Q1H)
Resolution range	42.22 - 1.75 (1.81 - 1.75)	49.60 - 1.35 (1.398-1.35)
Space group	$P\ 2_1$	$P\ 2_1\ 2_1\ 2_1$
Unit cell	50.97, 52.39, 72.87; 90, 101.80, 90	25.81, 52.46, 152.12; 90, 90, 90
Unique reflections	48567 (5071)	46366 (4424)
Multiplicity	6.9 (7.2)	2.0 (2.0)
Completeness (%)	90.00 (95.91)	99.43 (97.34)
Mean I/sigma(I)	3.99 (1.30)	17.87 (2.02)
Wilson B-factor	15.58	13.46
R-merge	0.2215 (1.516)	0.02098 (0.3197)
R-pim	0.0900 (0.603)	0.02098 (0.3197)
CC1/2	0.995 (0.402)	0.999 (0.759)
Reflections used in refinement	34326 (3632)	46363 (4424)
R-work	0.2330 (0.3709)	0.1648 (0.1912)
R-free	0.2630 (0.3957)	0.1971 (0.2624)
Number of non-hydrogen atoms	3689	1834
macromolecules	3453	1754
solvent	236	80
Protein residues	465	229
RMS(bonds)	0.004	0.009
RMS(angles)	0.66	0.96

bioRxiv preprint doi: https://doi.org/10.1101/2025.08.18.670751; this version posted August 18, 2025. The copyright holder for this preprint (which was not certified by peer review) is the author/funder, who has granted bioRxiv a license to display the preprint in perpetuity. It is made available under aCC-BY 4.0 International license.

Ramachandran favored (%)	97.81	98.67
Ramachandran allowed (%)	2.19	1.33
Ramachandran outliers (%)	0.00	0.00
Average B-factor	22	18
macromolecules	22	16
solvent	25	22

Statistics for the highest-resolution shell are shown in parentheses.

Supplementary Table 2: Designed Protein Sequences

Target	Binder	Binder sequence
FimH	FimH minibinder F7	MAEKEAALTAAADGTVAALAAGNIGVDYARYRRKALVAYAKKEGLPQAVIDAVTARLDAAIAAAEAA
FimH	FimH minibinder C8	MEEKIKAAKEAADGTVAALAAGNIGVDYARYYKKALVAWMKKQGLPQEVIDEVTAKLDAAIAAAEAA
Abp1D, Abp2D	Abp minibinder A7	EKSYEEAVLEANKLIESGAPDEEVEKATKYALDKYAASIGLSVVEYPPLETLKEFVTKEAAKIRAA
Abp1D, Abp2D	Abp oligomer C11	MKVYEFPYPETGKKIIVIQGEKNIVIVVGNTAVVYYEGKWTYKENVTEEDIEKAKTEEGAKELAKGSGEKSYEEAVLEANKLIESGAPDEEVEKATKYALDKYAASIGLSVVEYPPLETLKEFVTKEAAKIRAA

bioRxiv preprint doi: https://doi.org/10.1101/2025.08.18.670751; this version posted August 18, 2025. The copyright holder for this preprint (which was not certified by peer review) is the author/funder, who has granted bioRxiv a license to display the preprint in perpetuity. It is made available under aCC-BY 4.0 International license.

Supplementary Table 3: K_d estimates of FimH minibinders. This table includes the kinetic profiles (K_d's to LAS and HAS) of FimH minibinders that displayed affinity for either the HAS or LAS.

Binder_ID	LAS - K_d (nM)	HAS - K_d (nM)
A3	5000	3700
A4	2000	2500
B1	266	N.D.
B2	634	N.D.
C10	300	N.D.
C3	437	N.D.
C5	2000	2200
C7	2000	N.D.
D9	3500	4700
E10	4300	4200
E8	N.D.	4800
F1	939	729
F4	149	N.D.
**F7**	**100**	**N.D.**
G12	132	N.D.
H9	198	N.D.

N.D.: Binding was not detected. The K_d estimate is larger than the highest screened concentration (5 μM).

Bold: The design in bold was selected for further characterization.

bioRxiv preprint doi: https://doi.org/10.1101/2025.08.18.670751; this version posted August 18, 2025. The copyright holder for this preprint (which was not certified by peer review) is the author/funder, who has granted bioRxiv a license to display the preprint in perpetuity. It is made available under aCC-BY 4.0 International license.

Supplementary Table 4. Target Protein Sequences.

Target Name	Strain ID	PDB ID	Sequence	Reference
Abp1D	A. baumannii ACICU	8dez	NCTLSKGFTTVDIPMTIGTIVVRPTDPIGTVLQKNTFTISPNNSTATCNRASDQITAALPLNYPVSSIGNNVYATNIPGIGIRLYREAFDSTDFSGYYPYKRSLTPNTTYTLSPGYFVMEVIKTAATTGSGALVAGRYSTYYVTGQQNRPFLTTTVLSSSPILIASS	Tamadonfar, Kevin O., Gisela Di Venanzio, Jerome S. Pinkner, et al. "Structure–Function Correlates of Fibrinogen Binding by Acinetobacter Adhesins Critical in Catheter-Associated Urinary Tract Infections." Proceedings of the National Academy of Sciences 120, no. 4 (2023): e2212694120. https://doi.org/10.1073/pnas.2212694120.
Abp2D	A. baumannii ACICU	8df0	YCTLSSGFTTVDISMAVGRVVVRPSDPVGKILRKATFPINPNGSTLRCTSYSDTITAALTQNYPLSPLGNSIYSTNIPGIGIRLYREAENATNFSGYYPYTRSLTPGTTYNLAQGYFVVEIVKTADQTGSGTLVPGLYSRYYVNGHMDRPFLTSTVYGNAITIASSSHHHHHH	Tamadonfar, Kevin O., Gisela Di Venanzio, Jerome S. Pinkner, et al. "Structure–Function Correlates of Fibrinogen Binding by Acinetobacter Adhesins Critical in Catheter-Associated Urinary Tract Infections." Proceedings of the National Academy of Sciences 120, no. 4 (2023): e2212694120. https://doi.org/10.1073/pnas.2212694120
FimH_WT	E. coli K12	3jwn	FACKTANGTAIPIGGGSANVYVNLAPAVNVGQNLVVDLSTQIFCHNDYPETITDYVTLQRGSAYGGVLSSFSGTVKYNGSSYPFPTTSETPRVVYNSRTDKPWPVALYLTPVSSAGGVAIKAGSLIAVLILRQTNNYNSDDFQFVWNIYANNDVVVPT	Hvorecny, et al., (2025) Antibodies disrupt bacterial adhesion by ligand mimicry and allosteric interference. BioRxiv doi: https://doi.org/10.1101/2024.12.06.627246

bioRxiv preprint doi: https://doi.org/10.1101/2025.08.18.670751; this version posted August 18, 2025. The copyright holder for this preprint (which was not certified by peer review) is the author/funder, who has granted bioRxiv a license to display the preprint in perpetuity. It is made available under aCC-BY 4.0 International license.

FimH_L34K	E. coli K12	N/A	FACKTANGTAIPIGGGSANVYV NLAPAVNVGQNKVVDLSTQIFC HNDYPETITDYVTLQRGSAYGG VLSSFSGTVKYNGSSYPFPTTSE TPRVVYNSRTDKPWPVALYLTP VSSAGGVAIKAGSLIAVLILRQT NNYNSDDFQFVWNIYANNDVV VPT	Magala, et al. (2025) Ultra-slow conformational dynamics and catch bond formation of a Bacterial Adhesin revealed by a single-domain variant of FimH. BioRxiv doi: https://doi.org/10.1101/2025.07.17.665197
FimH_ST73	E. coli ST73	N/A	MKRVITLFAVLLMGWSVNAWS FACKTANGTAIPIGGGSANVYV NLAPAVNVGQNLVVDLSTQIFC HNDYPETITDYVTLQRGSAYGG VLSSFSGTVKYNGSSYPFPTTSE TPRVVYNSRTDKPWPVALYLTP VSSAGGVAIKAGSLIAVLILRQT NNYNSDDFQFVWNIYANNDVV VPTGGCDVSARDVTVTLPDYP GSVPIPLTVYCAKSQNLGYYLS GTTADAGNSIFTNTASFSPAQGV GVQLTRNGTIIPANNTVSLGAVG TSAVSLGLTANYARTGGQVTAG NVQSIIGVTFVYQ	https://www.ncbi.nlm.nih.gov/nuccore/NZ_UNQZ01000030.1
FimH_ST95	E. coli ST95	N/A	MKRVITLFAVLLMGWSVNAWS FACKTANGTAIPIGGGSANVYV NLAPVVNVGQNLVVDLSTQIFC HNDYPETITDYVTLQRGSAYGG VLSNFSGTVKYSGSSYPFPTTSE TPRVVYNSRTDKPWPVALYLTP VSSAGGVAIKAGSLIAVLILRQT NNYNSDDFQFVWNIYANNDVV VPTGGCDVSARDVTVTLPDYP GSVPIPLTVYCAKSQNLGYYLS GTTADAGNSIFTNTASFSPAQGV GVQLTRNGTIIPANNTVSLGAVG TSAVSLGLTANYARTGGQVTAG NVQSIIGVTFVYQ	https://www.ncbi.nlm.nih.gov/nuccore/CP043950.1

bioRxiv preprint doi: https://doi.org/10.1101/2025.08.18.670751; this version posted August 18, 2025. The copyright holder for this preprint (which was not certified by peer review) is the author/funder, who has granted bioRxiv a license to display the preprint in perpetuity. It is made available under aCC-BY 4.0 International license.

FimH_ST69	E. coli ST69	N/A	MKIICRLLLAMACLCLANISWA TVCANSTGVAEDEHYDLSNVF NSTNNQPGQIVVLPEKSGWVG VSAICPPGTLVNYTYRSYVTNFI VQETIDNYKYMQLNDYLLGAM SLVDSVMDIQFPPQNYIRMGTD PNVSQNLPFGVMDSRLIFRLKVI RPFINMVEIPRQVMFTVYVTST PNDPLVTPVYTISFGGRVEVPQN CELNAGQIVEFDFGDIGASLFSA AGPGNRPAGVMPQTKSIAIKCT NVAAQAYLTMRLEASAVSGQA MVSDNQDLGFIVADQNDTPITP NDLNSVIPFRLDAAAAANVTLR AWPISITGQKPTEGPFSALGYLR VDYQ	https://www.ncbi.nlm.nih.gov/nuccore/CP102488.1
FimH_ST131-H30	E. coli ST131-H30	N/A	MKRVITLFAVLLMGWSVNAWS FACKTANGTAIPIGGGSANVYV NLAPAVNVGQNLVVDLSTQIFC HNDYPETITDYVTLQRGSAYGG VLSNFSGTVKYSGSSYPFPTTSE TPRVVYNSRTDKPWPVALYLTP VSSAGGVAIKAGSLIAVLILRQT NNYNSDDFQFVWNIYANNDVV VPTGGCDVSAHDVTVTLPDYP GSVPIPLTVYCAKSQNLGYYLS GTTADAGNSIFTNTASFSPAQGV GVQLTRNGTIIPANNTVSLGAVG TSAVSLGLTANYARTGGQVTAG NVQSIIGVTFVYQ	Chen SL, Hung CS, Pinkner JS, Walker JN, Cusumano CK, Li Z, Bouckaert J, Gordon JI, Hultgren SJ. Positive selection identifies an in vivo role for FimH during urinary tract infection in addition to mannose binding. Proc Natl Acad Sci U S A. 2009 Dec 29;106(52):22439-44. doi: 10.1073/pnas.0902179106. Epub 2009 Dec 16. PMID: 20018753; PMCID: PMC2794649.
FimH_Klebsiella	K. pneumoniae (E. coli cas665)	N/A	MMKKIIPLFTTLLLGWSMNAW SFACKTATGATIPIGGGSANVYV NLTPAVNVGQNLVVDLSTQIFC HNDYPETITDYVTLQRGAAYG GVLSSFSGTVKYNGTSYPFPTT TETARVIYDSRTDKPWPAVLYLT PVSTAGGVAITAGSLIAVLILHQT NNYNSDSFQFIWNIYANNDVVV PTGGCDVSARDVTVTLPDYPGS MAVPLTVHCAQSQQLGYYLSG TTADSANAIFTNTASASPAQGIG VQLTRNGSAVPANSTVSLGTVG TSPVNLGLTATYARTTGQVTAG	Stahlhut SG, Chattopadhyay S, Struve C, Weissman SJ, Aprikian P, Libby SJ, Fang FC, Krogfelt KA, Sokurenko EV. Population variability of the FimH type 1 fimbrial adhesin in Klebsiella pneumoniae. J Bacteriol. 2009 Mar;191(6):1941-50. doi: 10.1128/JB.00601-08. Epub 2009 Jan 16. PMID: 19151141; PMCID: PMC2648365.

bioRxiv preprint doi: https://doi.org/10.1101/2025.08.18.670751; this version posted August 18, 2025. The copyright holder for this preprint (which was not certified by peer review) is the author/funder, who has granted bioRxiv a license to display the preprint in perpetuity. It is made available under aCC-BY 4.0 International license.

			NVQSIIGITFVYQ	

Supplementary Table 5: K_d estimates of Abp minibinders. This table includes the kinetic profiles (K_ds to Abp1D and Abp2D) of Abp minibinders that were enriched during yeast surface display and expressed sufficiently for downstream characterization as measured by SPR. Minibinders that exhibited undetectable binding to both Abp1D and Abp2D were excluded for clarity.

**Binder_ID**	**Abp1D - K_d (nM)**	**Abp2D - K_d (nM)**
**A4**	**40**	**559**
A6	3900	P.F.
**A7**	**67**	**4**
A8	2030	P.F.
A12	N.D.	235
B1	P.F.	48
B4	N.D.	786
B5	987	1010
B6	1260	336
**B7**	**101**	**16**
B8	3830	201
B9	669	187
B12	N.D.	657
C1	1240	624
C6	N.D.	950
**C7**	**6**	**124**

bioRxiv preprint doi: https://doi.org/10.1101/2025.08.18.670751; this version posted August 18, 2025. The copyright holder for this preprint (which was not certified by peer review) is the author/funder, who has granted bioRxiv a license to display the preprint in perpetuity. It is made available under aCC-BY 4.0 International license.

**N.D.**: Binding was not detected. The K_d estimate is larger than the highest screened concentration (5 μM).
**P.F.**: The binding data could not be fit well to accurately estimate a K_d.
**Bold**: The designs in **bold** exhibited K_d's to both Abp1D and Abp2D at or below 1 μM.

bioRxiv preprint doi: https://doi.org/10.1101/2025.08.18.670751; this version posted August 18, 2025. The copyright holder for this preprint (which was not certified by peer review) is the author/funder, who has granted bioRxiv a license to display the preprint in perpetuity. It is made available under aCC-BY 4.0 International license.

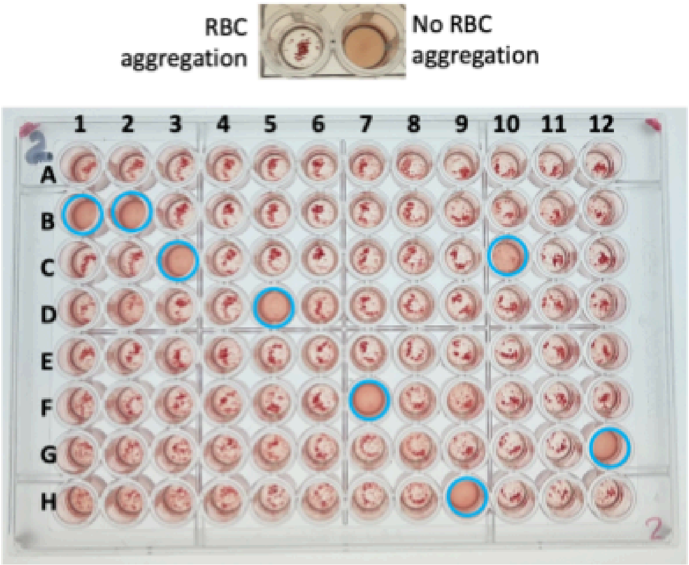

Figure S1: Initial RBC screen of designed FimH minibinders. Blue circles indicate binders that inhibit red blood cell aggregation.

bioRxiv preprint doi: https://doi.org/10.1101/2025.08.18.670751; this version posted August 18, 2025. The copyright holder for this preprint (which was not certified by peer review) is the author/funder, who has granted bioRxiv a license to display the preprint in perpetuity. It is made available under aCC-BY 4.0 International license.

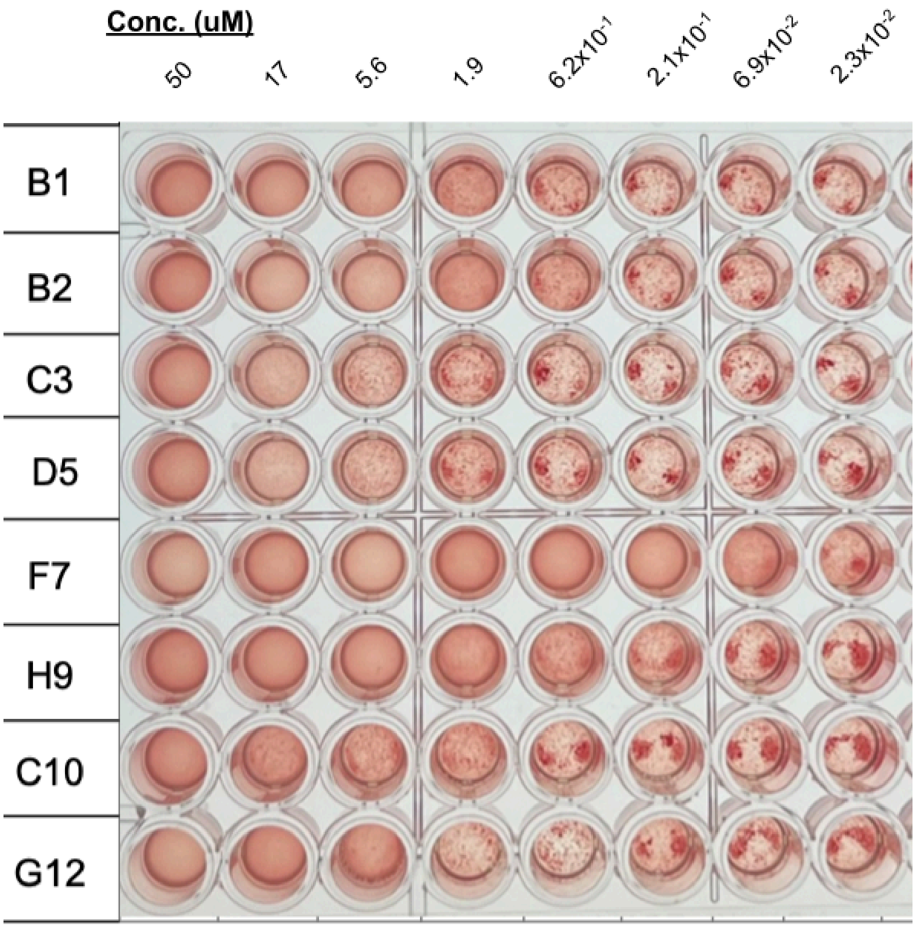

Figure S2: RBC inhibition titration of initial hits. Concentrations shown in micromolar for all minibinders identified as inhibitory in the initial screen.

bioRxiv preprint doi: https://doi.org/10.1101/2025.08.18.670751; this version posted August 18, 2025. The copyright holder for this preprint (which was not certified by peer review) is the author/funder, who has granted bioRxiv a license to display the preprint in perpetuity. It is made available under aCC-BY 4.0 International license.

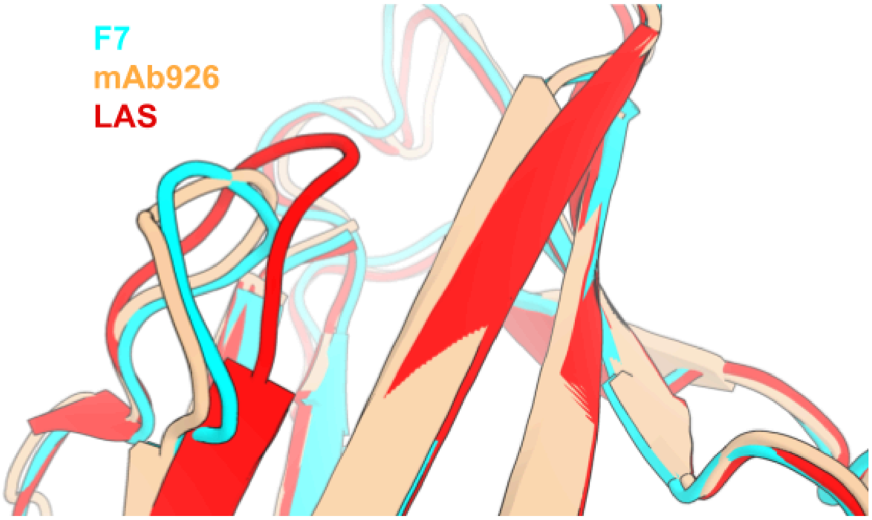

Figure S3: Cartoon overlay of FimH clamp loop. The fimH clamp loop is shown in the F7-FimH complex (cyan), mAb926-FimH complex (PDB code 9ME5; wheat), or apo-LAS (PDB code 3JWN; red).

bioRxiv preprint doi: https://doi.org/10.1101/2025.08.18.670751; this version posted August 18, 2025. The copyright holder for this preprint (which was not certified by peer review) is the author/funder, who has granted bioRxiv a license to display the preprint in perpetuity. It is made available under aCC-BY 4.0 International license.

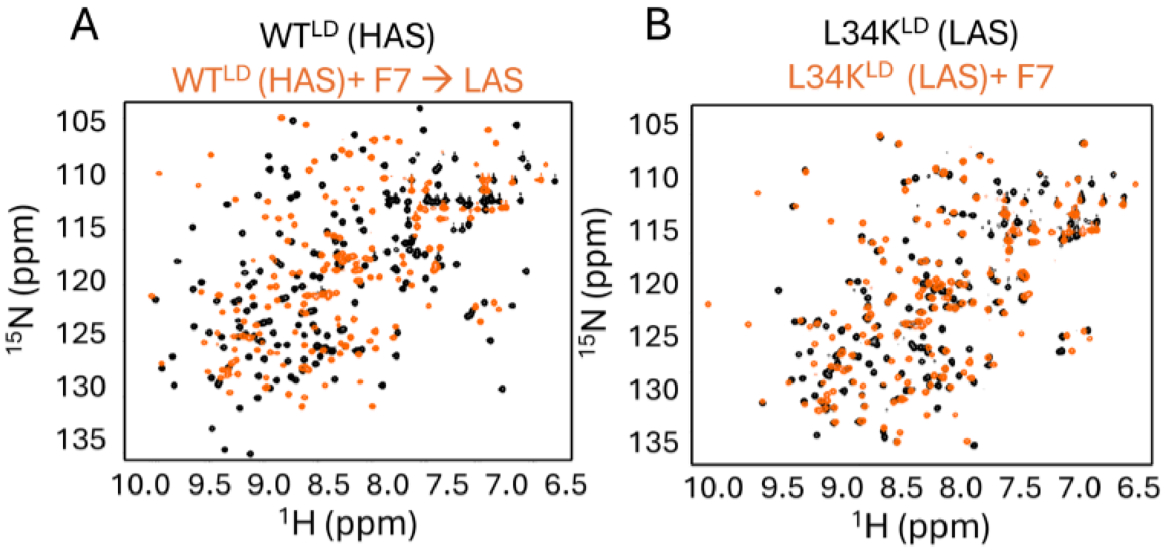

Figure S4: Overlay of FimH NMR spectra in presence and absence of minibinder F7. A) ^{15}N-HSQC NMR Spectra of FimH HAS (black) and HAS in the presence of minibinder F7 (orange). Note that the spectra shift markedly upon addition of F7. **B)** ^{15}N-HSQC NMR Spectra of FimH LAS (black) and LAS in the presence of minibinder F7 (orange). Note that the spectra remain largely unchanged upon addition of F7.

bioRxiv preprint doi: https://doi.org/10.1101/2025.08.18.670751; this version posted August 18, 2025. The copyright holder for this preprint (which was not certified by peer review) is the author/funder, who has granted bioRxiv a license to display the preprint in perpetuity. It is made available under aCC-BY 4.0 International license.

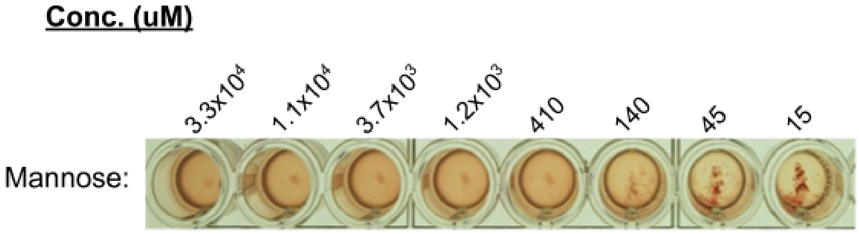

Figure S5: RBC inhibition titration of mannose only. The minimum inhibitory concentration of mannose on RBC aggregation is 140μM, several thousand fold higher than F7 (69 nM).

bioRxiv preprint doi: https://doi.org/10.1101/2025.08.18.670751; this version posted August 18, 2025. The copyright holder for this preprint (which was not certified by peer review) is the author/funder, who has granted bioRxiv a license to display the preprint in perpetuity. It is made available under aCC-BY 4.0 International license.

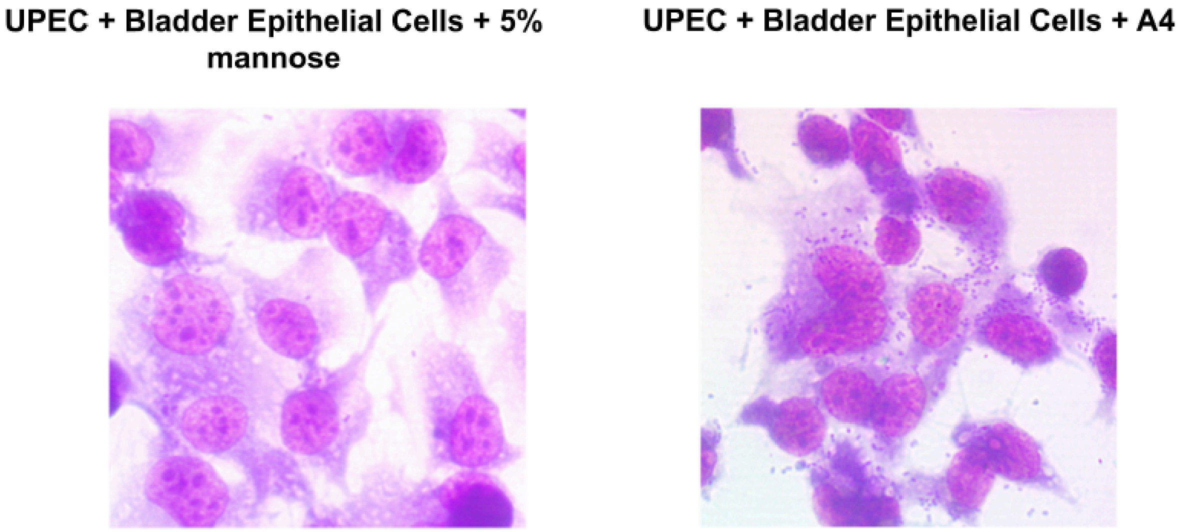

Figure S6: Confocal microscopy image of the inhibition of UPEC adhesion to bladder epithelial cells. Addition of high levels of mannose successfully inhibits colonization, depleting bacteria similarly to F7 (left). Addition of noninhibitory minibinder A4, meanwhile, fails to prevent bacterial colonization (right).

bioRxiv preprint doi: https://doi.org/10.1101/2025.08.18.670751; this version posted August 18, 2025. The copyright holder for this preprint (which was not certified by peer review) is the author/funder, who has granted bioRxiv a license to display the preprint in perpetuity. It is made available under aCC-BY 4.0 International license.

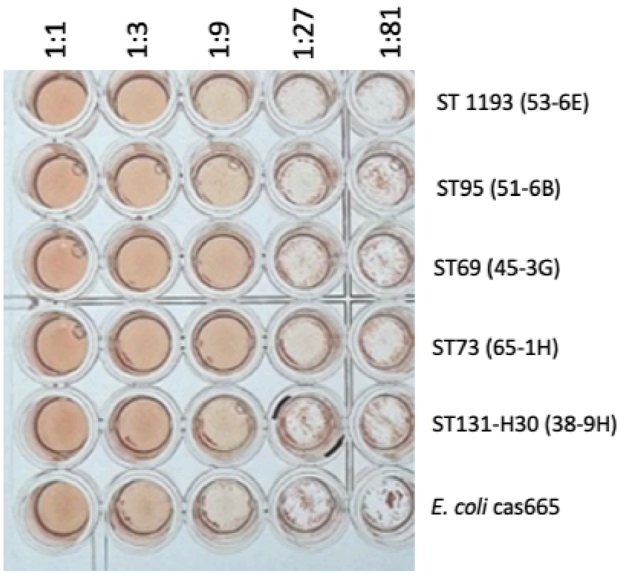

Figure S7: RBC inhibition titration of F7 against clinically relevant strains. F7 inhibition of RBC aggregation caused by clinical *E. coli* strains from the clonal groups (STs) of the major clinical importance and *E. coli* cas665 expressing *K. pneumoniae* FimH.

bioRxiv preprint doi: https://doi.org/10.1101/2025.08.18.670751; this version posted August 18, 2025. The copyright holder for this preprint (which was not certified by peer review) is the author/funder, who has granted bioRxiv a license to display the preprint in perpetuity. It is made available under aCC-BY 4.0 International license.

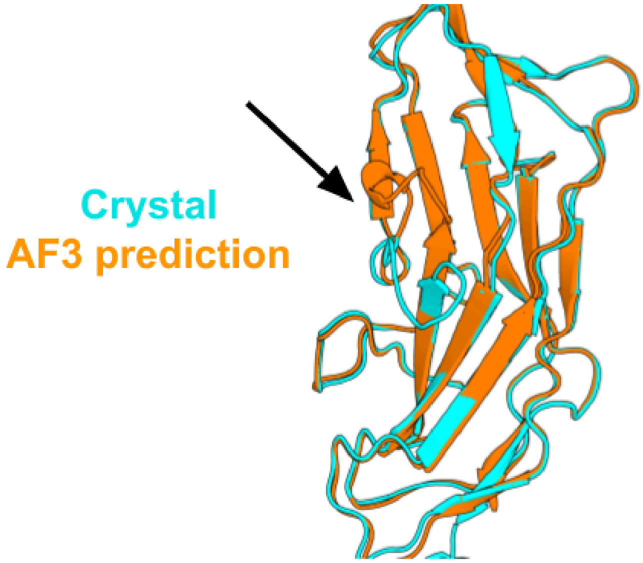

Figure S8: Cartoon overlay between the crystal structure of Abp2D and its Alphafold2 model. The arrow identifies the flexible anterior binding loop of Abp2D that is part of its putative fibrinogen binding pocket.

bioRxiv preprint doi: https://doi.org/10.1101/2025.08.18.670751; this version posted August 18, 2025. The copyright holder for this preprint (which was not certified by peer review) is the author/funder, who has granted bioRxiv a license to display the preprint in perpetuity. It is made available under aCC-BY 4.0 International license.

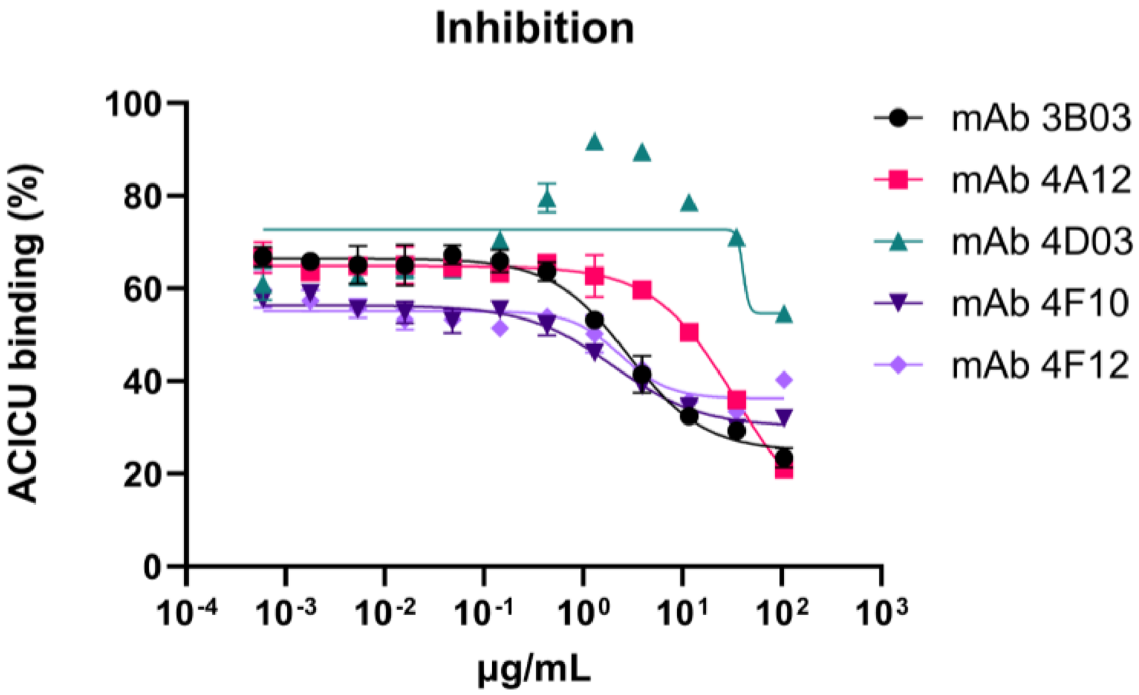

Figure S9: Purified adhesin ELISA for noninhibitory Abp2D mAbs. ELISA results for mAb-mediated inhibition of purified Abp2D binding to fibrinogen. The data provided contains 3 replicates. Error bars show standard deviation.

bioRxiv preprint doi: https://doi.org/10.1101/2025.08.18.670751; this version posted August 18, 2025. The copyright holder for this preprint (which was not certified by peer review) is the author/funder, who has granted bioRxiv a license to display the preprint in perpetuity. It is made available under aCC-BY 4.0 International license.

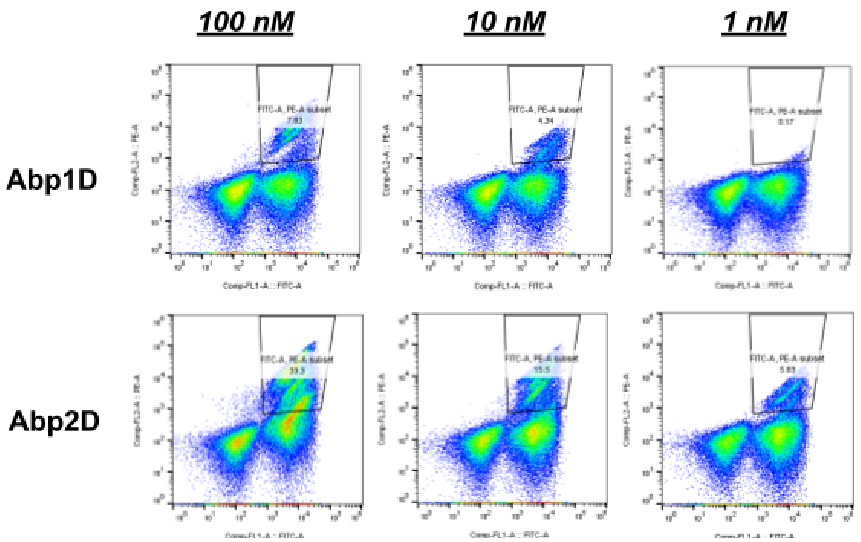

Figure S10: Titration sort results for yeast library and Abps. FACS results for sort 3 of the yeast display experiment using 10-fold titrations of Abp1D and Abp2D.

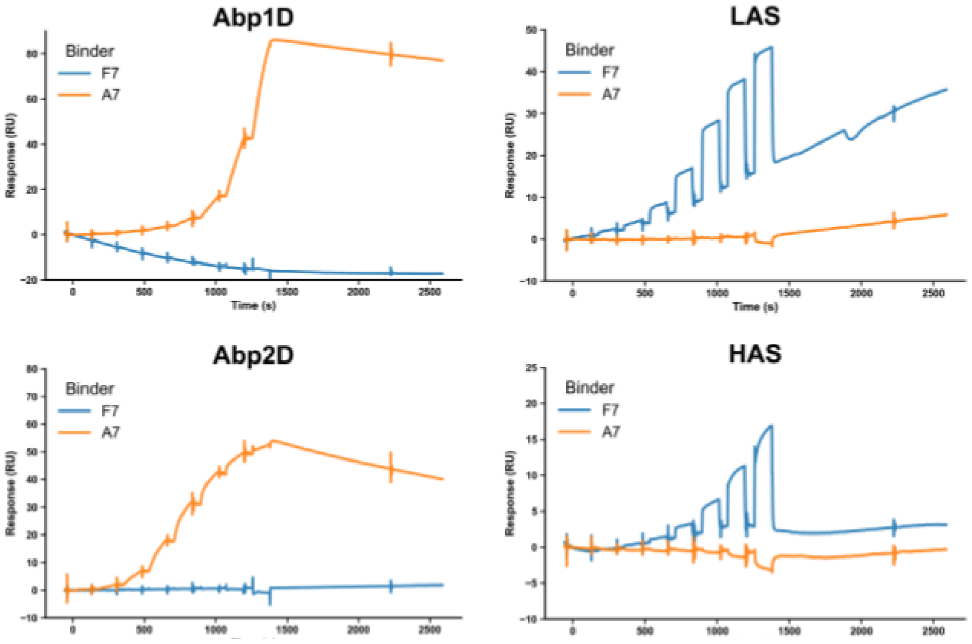

Figure S11: ***In vitro*** **specificity test for adhesin minibinders.** SPR traces for Abp minibinder A7 and FimH minibinder F7 with Abp1D, Abp2D, FimH LAS, and FimH HAS.

bioRxiv preprint doi: https://doi.org/10.1101/2025.08.18.670751; this version posted August 18, 2025. The copyright holder for this preprint (which was not certified by peer review) is the author/funder, who has granted bioRxiv a license to display the preprint in perpetuity. It is made available under aCC-BY 4.0 International license.

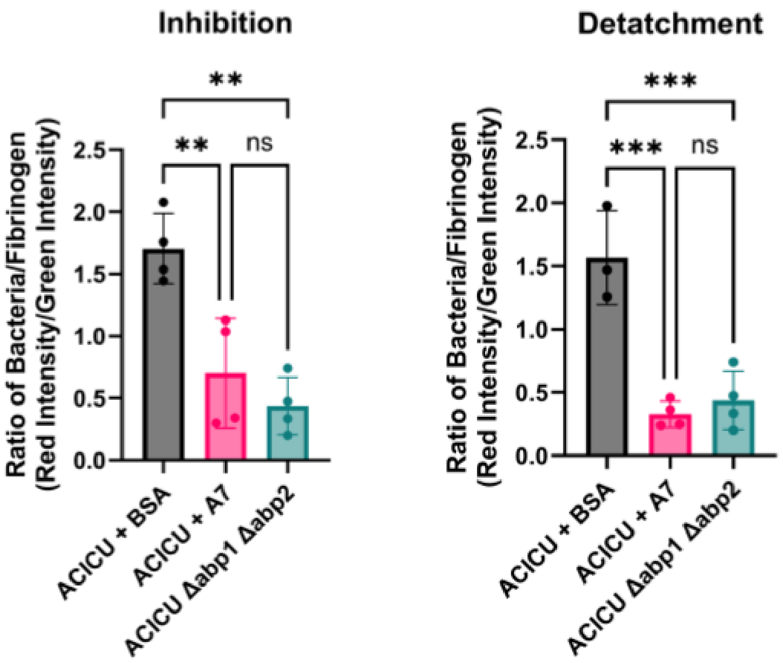

Figure S12: Quantification of *A. baumannii* binding to treated catheters a) preincubated (left) with 100 nM of A7 inhibitor or b) applied to catheters after bacterial attachment (right). Normalized signal intensity of *A. baumannii* bacteria (red signal) over fibrinogen coating (green signal) per catheter. n=4 for all groups, except for 100 nM BSA detachment where n=3. Error bars show standard deviation. One-way ANOVA test. ***P ≤ 0.01, **P ≤ 0.01, *P ≤ 0.05.

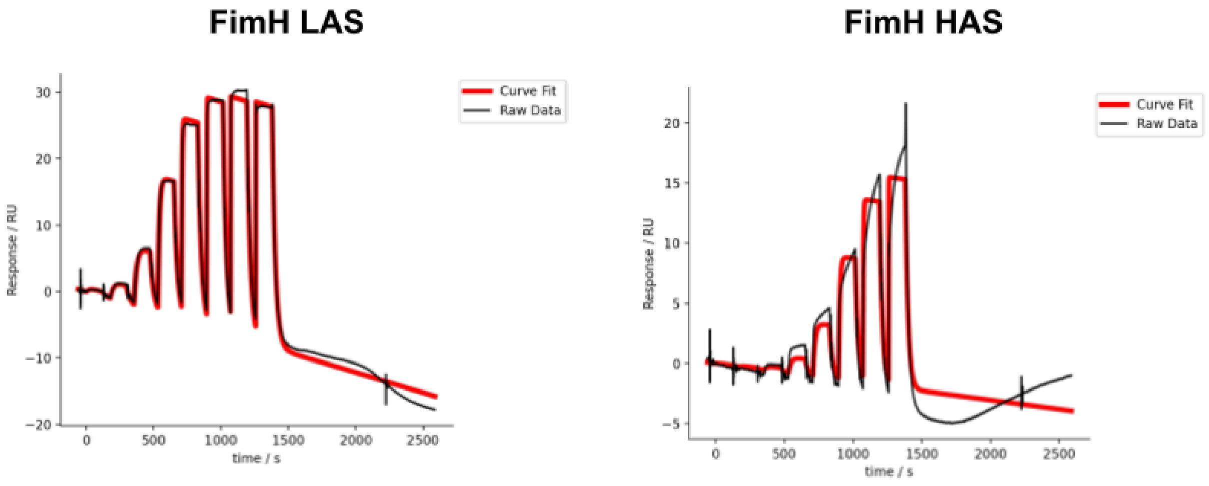

Figure S13: SPR Traces of FimH minibinder C8. Minibinder C8 binds with a higher affinity than F7 to both FimH LAS (K_d=15.0 nM) and FimH HAS (K_d=243 nM).

bioRxiv preprint doi: https://doi.org/10.1101/2025.08.18.670751; this version posted August 18, 2025. The copyright holder for this preprint (which was not certified by peer review) is the author/funder, who has granted bioRxiv a license to display the preprint in perpetuity. It is made available under aCC-BY 4.0 International license.

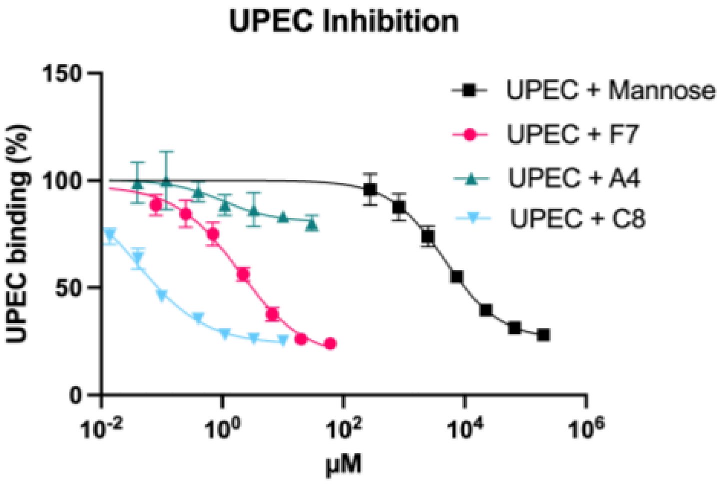

Figure S14: Inhibition ELISA showing minibinder C8. Inhibition ELISA results for minibinder F7 (pink; IC50=1.9 μM; 95% CI: 1.4-2.9 μM), noninhibitory minibinder A4 (teal), improved minibinder C8 (cyan; IC50-37 nM; 95% CI: 31-47 nMand mannose (black). Each experiment includes three replicates. Error bars show standard deviation.

bioRxiv preprint doi: https://doi.org/10.1101/2025.08.18.670751; this version posted August 18, 2025. The copyright holder for this preprint (which was not certified by peer review) is the author/funder, who has granted bioRxiv a license to display the preprint in perpetuity. It is made available under aCC-BY 4.0 International license.

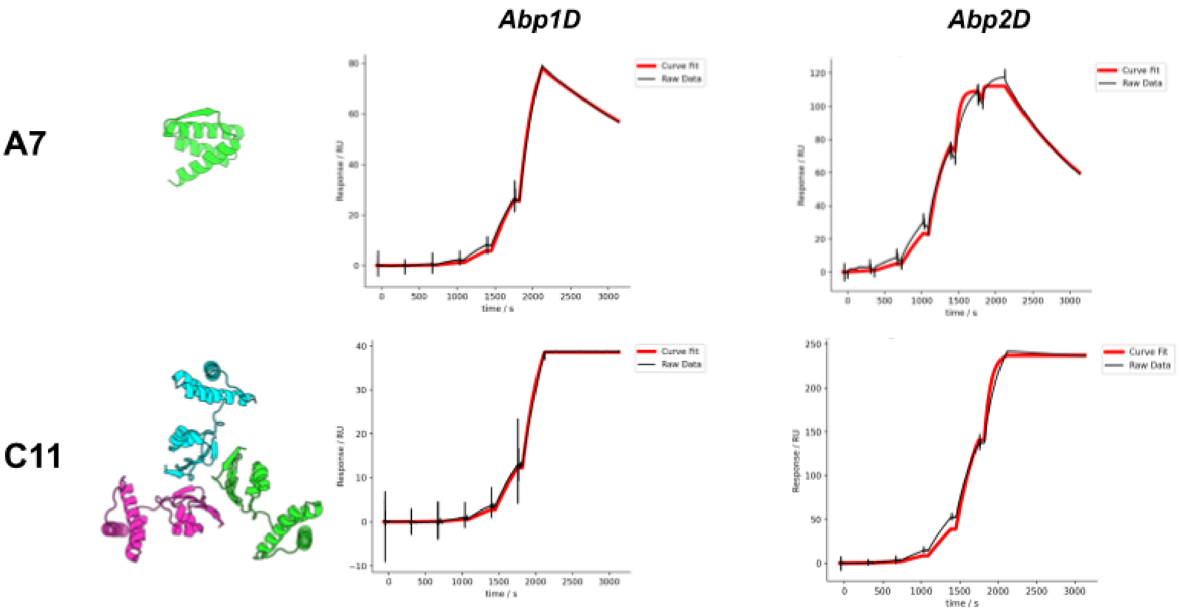

Figure S15: SPR traces of Abp Oligomer C11 and its parent: Abp minibinder A7. Oligomer C11 binds with a higher affinity than its parent to both Abp1D (K_d=11 pM) and Abp2D (K_d=195 pM).

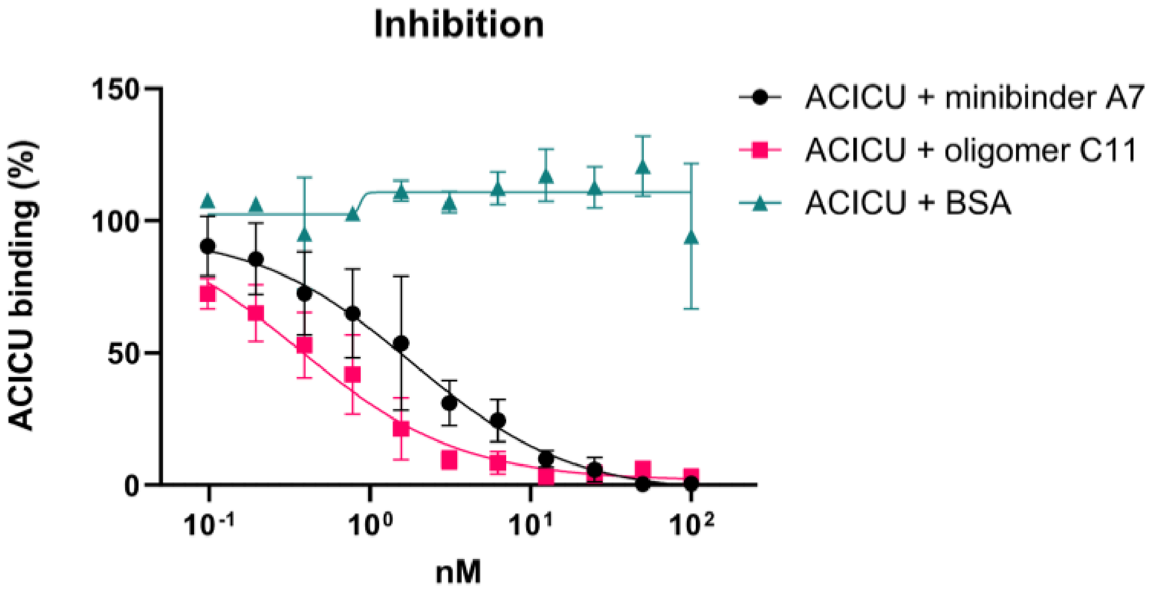

Figure S16: Inhibition ELISA results for Abp oligomer C11 and its parent: Abp minbinder A7. ELISA results indicate that oligomerization improves the IC50 of inhibition of fibrinogen binding *in cellulo*. The data provided contains 3 replicates. Error bars show standard deviation.
